# Crosstalk between endoplasmic reticulum stress and oxidative stress: a dynamic duo in multiple myeloma

**DOI:** 10.1007/s00018-021-03756-3

**Published:** 2021-02-18

**Authors:** Sinan Xiong, Wee-Joo Chng, Jianbiao Zhou

**Affiliations:** 1 Department of Medicine, Yong Loo Lin School of Medicine, National University of Singapore, Singapore, 117597 Republic of Singapore; 2 Centre for Translational Medicine, Cancer Science Institute of Singapore, National University of Singapore, 14 Medical Drive, Singapore, 117599 Republic of Singapore; 3 Department of Hematology-Oncology, National University Cancer Institute of Singapore (NCIS), The National University Health System (NUHS), 1E, Kent Ridge Road, Singapore, 119228 Republic of Singapore

**Keywords:** Multiple myeloma, Endoplasmic reticulum stress, Oxidative stress, Reactive oxygen species, Unfolded protein response

## Abstract

Under physiological and pathological conditions, cells activate the unfolded protein response (UPR) to deal with the accumulation of unfolded or misfolded proteins in the endoplasmic reticulum. Multiple myeloma (MM) is a hematological malignancy arising from immunoglobulin-secreting plasma cells. MM cells are subject to continual ER stress and highly dependent on the UPR signaling activation due to overproduction of paraproteins. Mounting evidence suggests the close linkage between ER stress and oxidative stress, demonstrated by overlapping signaling pathways and inter-organelle communication pivotal to cell fate decision. Imbalance of intracellular homeostasis can lead to deranged control of cellular functions and engage apoptosis due to mutual activation between ER stress and reactive oxygen species generation through a self-perpetuating cycle. Here, we present accumulating evidence showing the interactive roles of redox homeostasis and proteostasis in MM pathogenesis and drug resistance, which would be helpful in elucidating the still underdefined molecular pathways linking ER stress and oxidative stress in MM. Lastly, we highlight future research directions in the development of anti-myeloma therapy, focusing particularly on targeting redox signaling and ER stress responses.

## Introduction

Multiple myeloma (MM) is a genetically complex and heterogeneous hematologic malignancy with an estimated worldwide 5 year prevalence of around 230,000 individuals [1]. It is characterized by malignant proliferation of highly secretory monoclonal plasma cells in the bone marrow. MM is a progressive disease, which is usually preceded by premalignant asymptomatic conditions, monoclonal gammopathy of undetermined significance (MGUS) or smoldering myeloma (SMM). Despite recent advancement in stem-cell transplantation, high-dose chemotherapy and novel therapies, MM remains an incurable disease with an overall 5 year survival rate of approximately 53.9% from 2010 to 2016 [2]. Most myeloma patients experience relapses that require additional therapies, underscoring the urgent need to understand the mechanistic drivers of therapeutic resistance to develop better interventions.

The accurate and durable maintenance of functional cellular proteome requires an intricate regulation of the protein homeostasis (proteostasis) network and has been associated with aging and diseases [3]. Very mild perturbation of proteostatic fine-tuning and related stress-response pathways can be sufficient to impact not only a myriad of cellular functions but also tissue integrity [4]. Extensive protein synthesis in MM cells is accompanied by adaptive alterations in metabolic pathways [5], and accumulation of unfolded or misfolded protein in the ER lumen that triggers unfolded protein response (UPR) to resolve ER stress and restore proper protein folding.

Secretory cells like plasma cells have been estimated to create 3–6 million disulfide bonds per minute, which leads to the production of a comparable amount of intracellular reactive oxygen species (ROS) [6]. ROS can act as a second messenger driving carcinogenesis, cancer progression and metastasis. The delicate balance between ROS generation and scavenging plays an important role in redox homeostasis which is intimately linked to proteostasis. However, excessively high level of ROS induces oxidative stress that promotes genomic instability, damage to membrane permeability and protein oxidative modification. UPR signaling is also regulated by redox-controlled reversible modifications. One example is thiol oxidation of ER molecular chaperons such as the binding immunoglobulin protein (BiP/Grp78) that facilitates the release of BiP from ER stress sensors and activates UPR [7]. Another example is cysteine sulfhydration of protein tyrosine phosphatase 1B (PTP1B) that promotes phosphorylation and activation of double-stranded RNA-activated protein kinase-like ER kinase (PERK), one of the major ER stress sensors [8]. In addition, ROS mediate cysteine sulfenylation of another ER stress sensor, inositol requiring enzyme 1α (IRE1α), which activates nuclear factor-E2-related factor-2 (Nrf2) to generate an antioxidative response [9].

## The role of ER stress in multiple myeloma and drug resistance

The ER is a specialized protein-folding factory where protein quality control mechanisms allow only properly folded and modified proteins to be transported out of the ER *en route* to other organelles or plasma membrane. Genetic profiling analysis revealed that approximately half of the MM patients harbor mutations affecting RNA processing, protein translation, proteostasis and UPR [10]. ER stress response is, therefore, regarded as the “Achilles heel” of MM [11, 12].

**Fig. 1:**
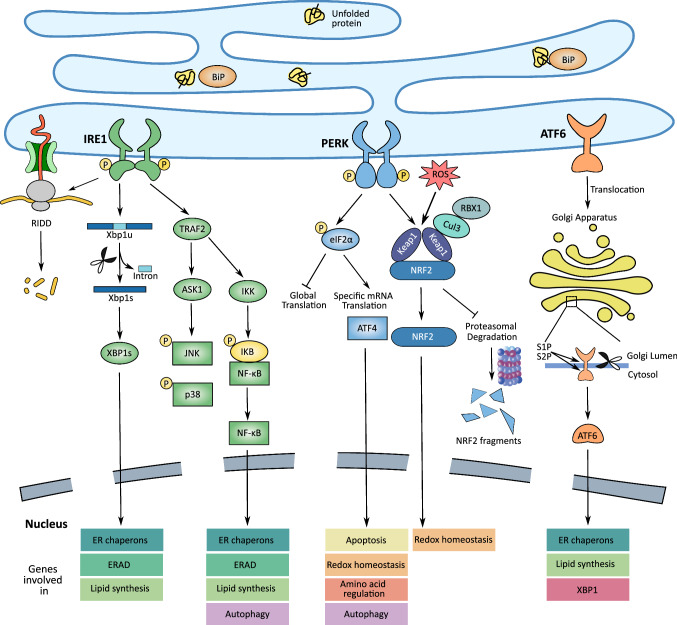
Signaling pathways associated with the UPR. To maintain ER homeostasis, accumulation of unfolded proteins that are bound by BiP in the ER activates three ER stress sensors, including IRE1, PERK and ATF6. However, chronic or excessive unresolved ER stress redirects the UPR pathways to trigger apoptosis. Dimerization and auto-phosphorylation of IRE1 induces its kinase and endoribonuclease activities, leading to phosphorylation of JNK and inhibitor of nuclear factor kappa B (IκB), unconventional splicing of XBP1 mRNA and RIDD. Similarly, dimerized PERK phosphorylates downstream targets eIF2α and NRF2 in the absence of BiP. On dissociation of BiP in the ER lumen, ATF6 translocates to the Golgi apparatus, where it undergoes cleavage by site-1 protease (S1P) and site-2 protease (S2P) to form the short-form ATF6 being redirected to the nucleus to mediate the expression of UPR downstream targets

The resolution of ER stress through UPR can be achieved in multifaceted ways by translational attenuation, cell cycle arrest, expansion of the ER compartment, upregulation of chaperon-mediated protein folding and refolding, and removal of aberrant proteins through ER-associated degradation (ERAD) and/or autophagy. The UPR signaling pathway engages three ER stress sensors, IRE1α, PERK and activating transcription factor 6 (ATF6) (Fig. 1). Under unstressed conditions, transmembrane protein IRE1, PERK, and ATF6 form complex with BiP/Grp78, thereby preventing IRE1 or PERK homodimerization or nuclear translocation of ATF6. Accumulation of unfolded protein triggers BiP/Grp78 release from these ER stress sensors and activates UPR signaling. In this section, we focus on the recent progress in recognizing the practical ramification and therapeutic significance of UPR signaling pathways in MM.

### IRE1α

Over the past decade, IRE1-mediated UPR, the most evolutionarily conserved signaling pathway in ER stress response, has been extensively studied for therapeutic potential in various types of cancers, including MM [13–16]. Activated IRE1α catalyzes the removal of an intron from the X-box binding protein 1 (XBP1) mRNA, leading to a translational frame-shift and production of an activated form of XBP1 [17] (Fig. 1). The spliced XBP1 induces transcriptional activation by modulating the expression of ER stress-responsive genes engaged in the ER membrane expansion, protein-folding machinery and ERAD, such as ER-resident chaperon p58IPK, BiP co-factor ERdj4, protein disulfide isomerase-P5 (PDI-P5) and ER degradation-enhancing alpha-mannosidase-like protein (EDEM) [18, 19]. XBP1 is frequently upregulated in MM cells and serves as a pro-survival factor that controls immunoglobulin production and inhibits apoptosis through activation of nuclear factor-κB (NF-κB) and activator protein-1 (AP-1) signaling pathways. Gupta et al. showed that XBP1 splicing is enhanced by heat-shock protein 70 kDa (HSP70) that protects cells from apoptosis under ER stress conditions. HSP70 also directly interacts with IRE1α and upregulates its endonuclease activity [20]. XBP1 splicing has been implicated in drug resistance in MM, which is in part associated with HSPs. In conferring a protective effect against bortezomib, MM cells upregulate expression of HSPs such as HSP27, HSP70 and HSP90 concomitantly with an increase in XBP1 activity [21]. Inhibition of IRE1α endonuclease domain or XBP1 splicing abrogates drug resistance in myeloma cells and increases sensitivity to proteasome inhibitors [15]. HSP70 and HSP90 inhibitors elicit similar effects with a negative impact on the stability of IRE1α and XBP1 [22, 23].

XBP1 has been proposed as an independent prognostic factor in MM. It was reported that low XBP1 spliced/unspliced ratio (XBP1s/XBP1u) is significantly correlated with improved overall survival and better clinical outcome in MM patients treated with immunomodulatory agent thalidomide [24]. In addition, higher level of total XBP1 mRNA expression predicts better clinical response in MM patients treated with bortezomib [25, 26]. Several groups have also shown that suppression of IRE1α-XBP1 signaling pathway contributes to acquired resistance to proteasome inhibitor (22–25). This is consistent with the observation that XBP1 knockdown weakens cytotoxicity of bortezomib, with XBP1s sensitizing cells to bortezomib [27]. Whole genome and exome sequencing of MM patients revealed two inactivating mutations in XBP1, P326R and L167I [10]. P326R, a missense mutation located within the transactivation domain of spliced XBP1 isoform has no effect on its transcription activity [28]. The other mutation, L167I is a splicing site mutation that impairs XBP1 signaling by reducing spliced XBP1 isoform while increasing the unspliced counterparts. Further research showed that XBP1-P326R and XBP1-L167I are unable to restore bortezomib sensitivity in XBP1 silenced-MM cells [27].

IRE1α can switch from the adaptive mode to pro-apoptotic mode through JNK-mediated apoptotic pathways and IRE1-dependent decay of mRNA (RIDD) [29]. Although RIDD is believed to inhibit ER stress-induced cell death by alleviating protein translocational load [30], prolonged activation of RIDD potentiates apoptosis through degradation of ER protein chaperone mRNAs and anti-apoptotic pre-microRNAs [31, 32]. Alternatively, IRE1 induces apoptosis via direct interaction with TNF receptor-associated factor 2 (TRAF2) and activates apoptosis signal-regulating kinase 1 (ASK1), followed by stimulation of C-Jun N-terminal kinase (JNK) and p38 signaling pathways [33, 34]. Oxidative stress also induces activation of ASK1 by dissociation of the ASK1-thioredoxin (TDX) complex in a ROS-dependent manner [35–37], indicating the presence of signaling connections between oxidative stress- and ER stress-induced apoptosis.

### PERK

PERK is an ER-resident kinase that has two main UPR-related substrates, eukaryotic initiation factor-2α (eIF2α) and Nrf2 (Fig. 1). In response to ER stress, activated PERK phosphorylates eIF2α at Ser51 and blocks the formation of pre-initiation complex, inducing a temporary halt in the protein translation concomitant with selective mRNAs translation of genes such as activating transcription factor 4 (ATF4) and C/EBP-homologous protein (CHOP/GADD153). ATF4 induces translational upregulation of the ER chaperon genes and antioxidative stress-response genes to facilitate stress adaption [38]. The eIF2α initiation factor induces ATF4-independent activation of protein kinase B (AKT) by downregulating mammalian target of rapamycin complex 1 (mTORC1) that exerts negative feedback regulatory effects on the oncogenic phosphoinositide 3-kinase (PI3K)-AKT signaling pathway [39]. In addition, PERK regulates antioxidative response and detoxification through phosphorylation and activation of Nrf2 [40].

PERK is required for malignant transformation and survival of MM cells. Michallet et al. reported that inhibition of PERK-eIF2α pathway in human MM cell lines exacerbates ER stress and promotes autophagic cell death due, at least in part, to the release of translational repression and amplification of the other two PERK-independent UPR pathways [41]. Interestingly, induction of autophagic pathway was found to suppress the mitochondrial apoptotic pathway in MM cells, indicating the role of PERK in metabolic regulation of plasma cell malignancy. This is corroborated by previous findings that PERK remains inactivated in association with ER chaperon p58IPK during normal plasma cell differentiation, whereas PERK expression is highly upregulated in myeloma cells [42, 43]. The probability of developing drug resistance increases with elevated resistance to ER stress. PERK offers a growth advantage to carfilzomib-resistant MM cells by upregulating pro-survival autophagy to overcome excessive accumulation of misfolded proteins [44]. Activation of PERK also promotes transcription of drug efflux transporters, such as multidrug resistance-related protein 1 (MRP1) and p-glycoprotein (P-GP), driven by Nrf2 [45]. The phosphorylation of eIF2α has been shown to mediate oxidative stress adaption in drug-resistant MM cells, rendered by enhanced cysteine transport and antioxidant glutathione (GSH) synthesis through activation of cystine/glutamate antiporter xCT (SLC7A11) [46, 47]. Therefore, targeting PERK-mediated regulatory axis is envisaged as a promising therapeutic strategy, but it is worth noting that inhibition of PERK may be accompanied by pancreatic toxicity.

However, PERK-eIF2α-ATF4 pathway has been shown to take part in a diametrically opposed function as a tumor suppressor. ATF4 may prompt cell death by inducing expression of genes encoding CHOP, growth arrest and DNA damage-inducible protein (GADD34) and ATF3, thereby overwhelming the cytoprotection mechanisms by cell death mediators [48]. Moreover, ATF4 interacts with CHOP to implement transcriptional regulation that increases protein synthesis, leading to ATP depletion and ROS accumulation [49]. Bortezomib was shown to evoke mitochondrial apoptosis in MM cells by upregulation of BH3-only proteins and activation of BAX/BAK [48, 50], following upregulation of pro-apoptotic terminal UPR signaling components including PERK and its downstream effectors ATF4 and CHOP [51]. Sustained eIF2α phosphorylation also exerts a death-promoting effect by activation of heme-regulated eIF2α kinase (HRI) in dexamethasone-resistant primary myeloma cells [52]. While current research fails to provide a clear-cut answer with regard to the underlying mechanisms of PERK signaling in controlling the UPR commitment to survival or death, emerging data suggest that cell fate decision under ER stress may be determined by the intensity and duration of UPR signal in concert with innate immune response and surrounding bone marrow microenvironment of MM cells [53, 54].

### ATF6

ATF6 is an ER transmembrane transcriptional activator of the ER stress-response element present in the promoters of various UPR-responsive genes, including the ER chaperon genes BiP, Grp94/Gp96, and P58^IPK^. The molecular mechanisms of ATF6 broadly overlap with the other ER stress sensors, although its role in MM remains largely unknown [55, 56]. Both ATF6 and PERK culminate in the transcription of CHOP involved with programmed cell death induced by anti-myeloma drugs [57–59]. In contrast, ATF6 exerts cytoprotective effects against otherwise detrimental ER stress and oxidative stress in concert with IRE1α [60, 61]. Specifically, ATF6 enhances XBP1 gene transcription, and splicing of XBP1 mRNA is mediated by IRE1α. Activation of both ATF6 and IRE1α are necessary to achieve maximal induction of the UPR, as implicated in the process of antibody-secreting plasma cell differentiation and maturation. ATF6 may also have a critical role in maintaining MM cell viability. A profound reduction in cell survival was observed in MM cells after knockdown of ATF6 [41]. Recently, ATF6 has also been proposed as a predictor of bortezomib sensitivity in MM cells since drug resistance evolves to reduce cellular dependence on the UPR by downregulation of ATF6 and XBP1 expression [62].

### UPR and plasma cell development

In addition to malignant transformation of plasma cells, normal plasma cell development also requires activation of UPR components, which is essential to accommodate increased demand on immunoglobin synthesis and counteract ER stress arising from B-cell differentiation process. Plasma cells play a central role in humoral antibody response and comprise at least two distinct populations: short-lived and long-lived plasma cells. Immature B cells migrate from the bone marrow to peripheral lymphoid organs, such as spleen and lymph nodes, where they achieve maturity and wait to be activated by foreign antigens. On encountering antigen, mature B cells rapidly differentiate into short-lived plasma cells (SLPC) that secrete predominantly IgM with low antigen-binding affinity in the peripheral lymphoid organs, while long-lived plasma cells (LLPC) residing primarily in bone marrow produce IgG with high stability and affinity, thus contributing to prolonged humoral immune response [63]. MM and LLPC share many common extrinsic and intrinsic survival mechanisms, underlining the importance of studying LLPC regulatory programs and the associated implications for MM disease progression and treatment. The bone marrow microenvironment provides critical extrinsic cues for survival and retention of MM and LLPC [64, 65]. For example, CD28 expression supports LLPC and MM survival and regulates immunoglobulin production via interaction with CD80/CD86-expressing stromal cells in the bone marrow niche [66, 67]. In addition, MM and LLPC carry intrinsic programs, including increased basal level of autophagy, glycolysis and responsiveness to ER stress, which sustain cell viability and ensure efficiency and fidelity of protein secretion in response to nutrient availability [68].

The number and longevity of plasma cells are tightly controlled over the course of humoral immune response against pathogens. Majority of plasma cells succumb to apoptosis after a few days of exuberant secretion of antibody. However, key apoptotic caspases (caspase-3/9) are subject to inactivation favoring SLPC survival and sustained high level of immunoglobulin secretion under conditions of ER stress in an early phase [69]. ER stress progressively becomes overwhelmed due to decreased proteasomal capacity, resulting in protein overload and caspase-independent cell death despite activation of the UPR [70, 71]. Hence, the coordinated regulation of caspase activity and ER stress is important in the temporal control of plasma cell death to ensure proper early humoral immune response.

The process of B cell to plasma cell differentiation involves extensive chromatin remodeling and epigenetic modification that elicit upregulation of master transcription regulators and reprogramming factors such as IRF4, Blimp-1, and XBP1. IRF4 is induced in response to NF-κB activation and is required for plasma cell survival partly through inhibition of caspase activation [72]. IRF4 does not appear to regulate key anti-apoptotic B-cell lymphoma 2 (Bcl-2) family protein myeloid cell leukemia 1 (Mcl-1), but overexpression of Bcl-2 can rescue cell death induced by loss of IRF4 in plasma cells. IRF4 also functions as a dose-dependent regulator of germinal center (GC) B-cell formation and plasma cell differentiation [73]. IRF4 is present at low levels in follicular B cells and transient expression of IRF4 stimulates initiation of GC formation. Signaling through B-cell antigen receptor (BCR) and engagement of co-stimulatory receptor on T cells leads to increased level of IRF4, which upregulates Blimp-1 and subsequently antagonizes the expression of Bcl6, a master regulator of the GC program. Hence, high levels of IRF4 induces B-cell exit from the GC reaction and reinforces plasma cell differentiation program. Notably, Blimp-1 shares overlapping functions with IRF4 in the regulation of UPR components and plasma cell identity. Specifically, Blimp-1 can induce the activation of ATF6 and its downstream effector XBP1, and also promote IRE1alpha-mediated XBP1 mRNA splicing [74, 75]. The downstream activation of XBP1 causes elevation of secretory pathway genes, ER expansion and lysosomal transport. In addition, Blimp-1 deficiency has been reported to attenuate immunoglobulin synthesis and secretion and decrease the activation of mTOR, without affecting the long-term survival of plasma cells [76].

UPR signaling branches are differentially activated during plasmacytic differentiation to optimize immunoglobulin production and secretion. Recent studies reveal that IRE1-XBP1 and ATF6 arms of the UPR are selectively activated upon lipopolysaccharide (LPS) stimulation of splenic B cells, whereas PERK-mediated signaling pathway is not induced after B-cell activation [77]. Reimold et al. reported that mice deficient in XBP1 have normal number of B cells but fail to generate antibody-secreting plasma cells [75], indicating an indispensable role of XBP1 in the terminal differentiation towards plasma cells. Indeed, XBP1 specifically regulates the synthesis of immunoglobulin μ heavy chains and ensures proper translocation of μ heavy chains to the ER [78]. Some studies have shown that expression of spliced XBP1 and cleavage of ATF6α precede the induction of Immunoglobulin synthesis and reach a maximum when antibody production is significantly induced, while other studies found that initial ER expansion and IgM production may occur prior to activation of XBP1 [55, 79]. ATF6 can also upregulate XBP1 expression through direct binding to its promoter, but it is believed that ATF6 alone is not sufficient to fully induce plasma cell differentiation as IRE1-mediated XBP1 mRNA splicing is required for activating XBP1 [80]. Furthermore, in contrast to the essential role of PERK in the normal development and secretory homeostasis of pancreatic β cells, PERK is dispensable for plasma cell development. Previous studies found no significant differences between wild-type and PERK^−/−^ B cells in terms of lifespan, viability as well as the ability to produce antibody in response to LPS [77, 81]. Although PERK is partially phosphorylated upon B-cell activation by LPS, it is not adequate to drive eIF2α phosphorylation and activate other downstream targets, such as CHOP, GADD34. It remains elusive how PERK arm of the UPR is inactive during plasma cell differentiation. One plausible explanation involves suppression of the PERK-mediated UPR by a negative regulator. Another possible mechanism is that, compared with IRE1 and ATF6, PERK may have a higher activation threshold in B cells. However, one study showed that ER stress inducers tunicamycin and thapsigargin exhibit comparable dose thresholds for the activation of XBP1 and CHOP [42].

### ER stress and autophagy

Normal and malignant plasma cells are highly reliant on the ubiquitin–proteasome system (UPS) and autophagy, which constitute the major protein degradation machineries and play critical roles in eliminating unfolded or misfolded proteins to sustain biosynthetic capability. The UPS is responsible for clearance of short-lived proteins and soluble unfolded or misfolded proteins, while insoluble, aggregation-prone proteins and organelles can be sequestered into autophagosome and subsequently degraded by autophagosome-lysosome fusion [82]. When proteasomal degradation is inhibited, the basal autophagy level is rapidly upregulated in MM cells to compensate for impaired proteasomal function, which can be beneficial for cell survival and overcoming drug-induced cytotoxicity. The adaptive response to proteasome stress entails increased expression of autophagic receptor/adaptor sequestosome 1 (SQSTM1)/p62 and altered interactome of SQSTM1/p62 that preferentially associate with ubiquitinated proteins [83, 84]. Recent studies have shown that SQSTM1/p62-mediated phase separation in autophagy regulates activation of the Nrf2-Keap1 signaling, thereby modulating redox homeostasis [85, 86]. Knockout of autophagy-related gene 5 (ATG5), an essential factor for autophagosome formation, gives rise to increased sensitivity to proteasome inhibitor MG-132, indicating the critical role of autophagy in regulating cellular response to proteasome inhibition [87]. Nevertheless, the clinical results of combined inhibition of proteasome and autophagic protein degradation have yielded contradictory results, which requires further delineation of the interconnection between UPS and autophagy [88, 89].

It is clear that both UPS and autophagy are involved in not only proteostasis but also amino acid homeostasis. In the absence of nutrient starvation, autophagy is maintained at low steady state levels, whereas UPS predominantly contributes to intracellular amino acid recycling. Autophagy can also be activated as an alternative mechanism for replenishment of the depleted amino acid pool in an attempt to alleviate amino acid scarcity induced by proteasome inhibition [90, 91]. The autophagy activation involves the PERK arm of the UPR and integrated stress response (ISR), which converge on eIF2α phosphorylation and result in upregulation of transcription factor ATF4-mediated amino acid deprivation response. ER stress has been shown to elevate expression of ATG12 and mediate microtubule‑associated protein light chain 3 (LC3) conversion during autophagosome formation via the PERK-eIF2α signaling pathway [92]. Overexpression of ATG5 and ATG7 can increase autophagic flux and suppress ER stress [93]. In addition, in amino acid-starved cells, the accumulation of uncharged tRNAs activates eIF2α kinase general control nonderepressible 2 (GCN2) that leads to induction of ISR signaling and alleviates mTORC1-mediated suppression of autophagy [94]. GCN2 activation is required for cell survival upon inhibition of proteasome and ATPase p97 (VCP/Cdc48), an essential component of ubiquitination machinery [95].

## Modulation of oxidative stress in multiple myeloma

In normal cells, redox homeostasis is maintained by elaborate regulation of ROS generation and antioxidant defense systems. Moderate increase in ROS has long been implicated in carcinogenesis and tumor progression, with functional effects on cellular proliferation, chromosomal instability and therapeutic resistance. However, excessive ROS production or impaired ROS scavenging capability at an unmanageable level can lead to oxidative stress and ultimate cellular death [96]. Endogenous ROS are mainly generated from mitochondria and peroxisomes. ER oxidative protein folding is another key source of ROS, particularly in secretory cells like antibody-producing plasma cells. The detrimental effects of ROS are counterbalanced by a comprehensive antioxidant defense systems comprising enzymatic antioxidants such as glutathione peroxidase (GPX), thioredoxin reductase (TrxR), catalase, peroxiredoxin (PRX) and superoxide dismutase (SOD), and non-enzymatic antioxidants such as GSH, thioredoxin (Trx), vitamin C, vitamin E, and metallothionein [97, 98].

Elevated level of ROS has been observed in numerous hematologic malignancies, and myeloma cells are no exception [99–101]. Studies have found that protein oxidation and lipid peroxidation are increased in MM patients when compared with healthy controls and MGUS patients, as measured by oxidative stress markers malondialdehyde (MDA) and advanced oxidation protein products (AOPPs) [102, 103]. Nevertheless, the antioxidant activities of SOD, GPX, catalase, and vitamins were found to be significantly lower in MM patients than in healthy controls [104]. Indeed, the abundance of intracellular antioxidants varies across different types of cancer. In patients with chronic leukemia, antioxidant parameters are elevated [105]; however, decline in antioxidant defense was observed in acute myeloid leukemia (AML) [99], pancreatic [106] and prostate cancer [107]. It is hypothesized that depletion of antioxidants may be due to increased scavenging efforts to counteract pro-oxidants and lipid peroxidation in cancer cells. It is also plausible that active oxidative molecules oxidize and inactivate enzymatic antioxidants such as GPX and SOD [108].

Notably, proteasome inhibitors have been shown to evoke oxidative stress-induced cell death through elevation of ROS level, oxidation of mitochondrial cardiolipin and loss of mitochondrial membrane potential [109]. The level of antioxidant defense has been considered as a critical factor in determining drug susceptibility in MM as antioxidant capacity is markedly elevated in drug-resistant cells to reduce oxidative stress [110, 111].

### Oxidative protein folding and ROS

Under ER stress conditions, the activity of ER chaperones and oxidoreductases increases as part of the effort to alleviate protein-folding burdens. The formation and isomerization of disulfide bonds, referred to as oxidative protein folding, are key steps in the post-translational modification of glycoprotein or secretory proteins [112]. This process involves electron transferring from reduced cysteine residues of the nascent proteins to protein disulfide isomerase (PDI) in an oxidized state. The resultant reduced PDI can be recycled by endoplasmic reticulum oxidoreductin-1 (ERO1) in a flavin adenine dinucleotide (FAD)-dependent reaction coupled with molecular oxygen reduction to hydrogen peroxide (H_2_O_2_), thus completing a catalytic turnover [113]. Oxidative protein folding is, therefore, considered as a significant contributor to ROS generation in MM cells with high basal ER stress, suggesting MM hypersensitivity to oxidative stress [114, 115].

ERO1 and PDI have multifaceted roles in cancer progression associated with tumor growth, angiogenesis and metastasis [116, 117]. High level of ERO1α expression was shown to be associated with poor overall survival rate in relapsed MM patients who received bortezomib or dexamethasone treatment [118]. ERO1 inhibitor EN-460 is sufficient to induce growth inhibition and apoptosis of MM cells, which is accompanied by exacerbation of ER stress due to upregulated ATF6- and eIF2α-mediated pathways. The ability of ERO1α to promote cancer cell proliferation is mediated by glycosylation and activation of cell surface receptor integrin-β1, specifically under hypoxic conditions [119]. Activation of ERO1α activity may enhance vascular endothelial growth factor (VEGF)-mediated angiogenesis in low-oxygen environment such as bone marrow where MM cells reside. Studies have found that MM cells secret high levels of VEGF that enhance the expression of interleukin-6 (IL-6) and Mcl-1, leading to persistent cell survival and proliferation [120, 121]. A recent study reported that MM cells resistant to bortezomib or carfilzomib constitutively express high level of PDI, whereas in drug-naïve parental cell line, PDI expression is strongly induced upon drug treatment [122]. Moreover, ER-resident peroxiredoxins IV (PRDX4) has a crucial role in the acceleration of oxidative protein folding by oxidation of PDI while catalyzing peroxide detoxification. PRDX4 is linked to immunoglobulin secretion, which is beneficial for B-cell differentiation and plasma cell malignant transformation [123].

Futile cycles of disulfide bond formation/isomerization and reduction driven by protein misfolding can lead to depletion of GSH, the most abundant antioxidant agent in the cells, thus further increasing ROS accumulation. GSH serves as a thiol/disulfide redox buffer that directly reduces improper disulfide pairings in proteins, allowing for another attempt of protein folding into the correct configuration [124, 125]. In addition, GSH reduces PDI and its homolog ERp57/Grp58, antagonizing oxidative stress by regulating disulfide bond formation rate [126]. The excessive consumption of GSH produces a copious amount of glutathione disulfide (GSSG) that exceeds the GSH recovery capacity governed by the glutathione reductase (GSH-Rd) and γ-glutamylcystein synthetase (GCS), ultimately leading to dysregulation of redox homeostasis. Of note, increased activities of GSH and GCS were observed in drug-resistant myeloma cell lines [127]. Further mechanistic study revealed that abrogation of bortezomib-induced apoptosis is partially mediated through inhibition of transcription factor Nrf2-associated downstream signaling that increases the GSH pool through upregulation of the xCT subunit of the Xc- cystine-glutamate antiporter [128]. A recent study revealed that FAM46C, one of the most recurrently mutated genes in MM, promotes ER expansion and augments immunoglobulin secretion, thus leading to a remarkable increase in oxidative stress that goes beyond the cellular capacity to maintain sustained proteostasis [129]. These findings are in line with previous research indicating that loss of oncosuppressive FAM46C confers a survival advantage to MM cells and poor prognosis in MM patients [130, 131]. Evasion of oxidative stress renders drug tolerance through several possible mechanisms including regulation of drug efflux by glutathione conjugation to xenobiotic compounds [132], suppression of p38 and JNK mitogen-activated protein kinase (MAPK) signaling pathways [133, 134] and removal of free radicals and lipid peroxides [135]. A recent genotyping study revealed the association between GST genetic polymorphism and MM susceptibility [136].

### NOX and ROS

NOX/dual oxidase (DUOX) is a family of transmembrane proteins that catalyze electron transfer from NADPH to molecular oxygen and generate ROS [137]. Mechanisms of NOX-dependent ROS generation have been previously reviewed [138, 139]. Seven isoforms of the NOX/DUOX enzyme family, which include NOX 1–5, DUOX1, and DUOX2, have been identified [140]. These isoforms exhibit differences in tissue distribution, intracellular localization, regulatory network and types of ROS generated. A large number of studies have elucidated the importance of NOX2-dependent ROS production in B-cell activation and differentiation [141–143]. NOX2 is the most abundant NOX/DUOX isoform in B lymphocytes, whereas other isoforms are expressed at very low or undetectable levels. Intriguingly, B cells from mice deficient in NOX2 or its catalytic subunit p47phox have severely impaired ROS-generating capability without alterations in the BCR signaling and antibody response following BCR stimulation [144, 145]. However, the finding that universal ROS scavenger N-acetylcysteine (NAC) inhibits BCR signaling indicates that NOX2 is involved in the early stage of BCR-dependent ROS production, whereas later stages of B-cell activation may require other sources of ROS, presumably mitochondria.

Increasing evidence points to the integral roles of NOX2 and NOX4 in the ER stress response, although their functional and clinical relevance in MM have not been extensively studied [146, 147]. Toll-like receptor (TLR)-dependent splicing of XBP1 mRNA is regulated by TRAF6 and NOX2. In MM, TLR4 activation contributes to cell proliferation and downregulation of CHOP and ATF4. NOX2-derived ROS are associated with the pro-apoptotic CHOP-Calcium/calmodulin-dependent protein kinase II (CaMKII) pathway, in which NOX2 promotes feed-forward amplification of CHOP through activation of double-stranded RNA-dependent protein kinase (PKR) and phosphorylation of its substrate eIF2α. On the other hand, NOX4-derived ROS inactivate protein tyrosine phosphatase 1B (PTP1B) that induces IRE1α- and eIF2α-mediated signaling pathways in response to ER stress [148, 149]. PTP1B oxidation has also been shown to elicit cellular senescence downstream of RAS [150], which may be related to myeloma pathogenesis since RAS is the single most frequently mutated oncogene in MM. Several studies have alluded to the tumor suppressive role of PTP1B in hematologic disorders, whereas its oncogenic role has been validated predominantly in solid tumor studies [151]. Dubé et al. reported that PTP1B deficiency in mice results in increased B cells in the bone marrow and lymph nodes, thus increasing the susceptibility to B lymphoma [152]. Le Sommer et al. reported that myeloid-specific PTP1B deficiency promotes cell survival and AML development in mice [153].

### Mitochondrial ROS

Mitochondria are important sources of ROS, metabolic byproducts resulted from electron leakage in the electron transport chain. Complex III is the major site of superoxide ^•^O_2_^−^ production under normal conditions while higher level of mitochondrial ROS is generated from complex I under many pathological conditions [154]. Mitochondrial ROS have garnered attention for playing a pivotal role in tumorigenesis. Complex III-derived ROS are required for hypoxia-inducible factor (HIF) stabilization during hypoxia, which increases glucose metabolism in glycolysis by upregulating the expression of glycolytic enzymes and glucose transporters, and shunts pyruvates away from oxidative metabolism by increasing pyruvate dehydrogenase kinase 1 (PDK1) [155]. Mitochondrial ROS have also been shown to oxidize and inactivate phosphatase and tensin homolog (PTEN), PTP1B and protein phosphatase 2A (PP2A), leading to activation of PI3K-AKT signaling pathway.

Mitochondria, as the central regulatory hub of Ca^2+^ homeostasis, are involved in absorption and buffering of cytosolic Ca^2+^, which is required for metabolic adaption to increased energy demand, and maintaining antioxidative capacity of reduced NADH and NADPH [156]. Unmitigated oxidative stress or ER stress may induce Ca^2+^ leakage from the ER and subsequent Ca^2+^ accumulation in the mitochondria, resulting in release of cytochrome c and second mitochondria-derived activator of caspase (smac) and ultimately activation of apoptotic signaling cascades [157]. Increased mitochondrial Ca^2+^ in turn collapses mitochondrial membrane potential (Δ*Ψ*_m_) and enhances ROS production through multiple mechanisms including increase in ubisemiquinone radicals due to blockade of electron transport at complex III, increase in O_2_ consumption by activation of Krebs cycle dehydrogenases, disruption of the electron transport chain by increased NO production and depletion of GSH pool [158]. This ROS-induced ROS-release process constitutes a positive feedback loop amplifying the cell death signals.

Recent studies, albeit limited, have shed light on the mitochondrial dependency for the development of MM [159, 160]. Intercellular transport of mitochondria from bone marrow stromal cells to MM cells was reported to render survival and proliferative advantage to MM cells via a CD38-dependent mechanism through elevation of oxidative phosphorylation and glycolysis [161]. It was believed that metabolic activities of MM cells rely primarily on glycolysis in the hypoxic bone marrow niche, while recent studies provide compelling evidence that the oxidative phosphorylation pathway is gradually upregulated in the process of MM development as well as in bortezomib-resistant MM cells. Single cell RNA sequencing analysis of CD138 + cells from 15 MM patients at different disease stages revealed that the expression of genes related to oxidative phosphorylation is lowest among MGUS patients and highest among patients with t(4;14) translocations [162]. Another interesting study showed that missense mutations in mitochondrial genes encoding components of complex I (MT-ND2, MT-ND4, and MT-ND5) and cytochrome c oxidase (MT-CO3) occur frequently in patients with relapsed MM, indicating the alterations of bioenergetics and metabolism in MM progression [163]. Consistent with these findings, previous studies found that mitochondrial dysfunction may confer drug resistance in MM cells characterized by Δ*Ψ*_m_ hyperpolarization, high basal mitochondrial Ca^2+^ level and oxygen consumption rate [164]. Hence, resistant MM cells may be more susceptible to drug-induced mitochondrial oxidative stress than their drug-naïve counterparts.

## Nrf2 as a nodal regulator of ER stress and oxidative stress

Oxidative insults can potentiate ER stress responses to neutralize ROS accumulation originated from excessive oxidative protein folding, mitochondrial respiration and detoxification. ROS direct sulfenylation of cysteine residues within the IRE1 kinase activation loop, inhibiting the IRE1-mediated UPR signaling and activating Nrf2 antioxidant stress responses [9]. The PERK-eIF2α-ATF4 pathway constitutes the integrated stress response that undergoes redox regulation, the activation of which is provoked by hypoxic conditions that prevent cells from ATP depletion through attenuation of global protein synthesis. A functional link between PERK and oxidative stress-induced PI3K-AKT pathway has been delineated [39, 165]. The eIF2α kinases PERK and general control non-derepressible-2 (GCN2) are required for AKT phosphorylation and activation, which are mediated by a negative feedback loop induced by mTORC leading to reduced eIF2α phosphorylation. Recently, ATF6 has been found to induce expression of antioxidant genes such as catalase, peroxiredoxin 5 (Prdx5) and valosin-containing protein-interacting membrane protein through increased gene transcription in response to oxidative stress [166].

Nrf2 is one of the key convergent points linking ER stress and oxidative stress. It is normally sequestered by microtubule-associated protein kelch‐like ECH‐associated protein 1 (Keap1) in the cytosol and targeted for degradation by the ubiquitination-proteasome machinery. Nuclear translocation of Nrf2 requires its dissociation from the Keap1-Cul3-RBX1 complex and inhibition of degradation by the ubiquitination-proteasome pathway, which typically occurs in response to oxidative stress, ER stress or xenobiotic exposure [167, 168]. Activated transcription factor Nrf2 regulates the intracellular redox homeostasis by inducing the expression of a repertoire of antioxidant and detoxifying genes, including GST [169], SOD [170], heme oxygenase-1 (HO-1) [40] and NAD(P)H:quinone oxidoreductase-1 (NQO1) [171], while reducing NOX expression [172]. The cysteine residues of Nrf2 are critical for sensing oxidant inducers and repressing Keap1-dependent degradation of Nrf2 [173]. Nrf2-Keap1 complex is localized at the outer mitochondrial membrane through interaction with phosphoglycerate mutase 5 (PGAM5) containing a mitochondrial-localization sequence [174]. This localization allows Nrf2 to sense mitochondrial ROS in vicinity and regulate stress defense. Various regulatory mechanisms of Nrf2 signaling have been described. These include cysteine oxidative modification of Keap1 that induces conformational change in Keap1, thus leading to Nrf2 release from Keap1 [175]. ROS can directly activate Nrf2 and influence protein turnover of Nrf2 through rapid induction of de novo synthesis of Nrf2. Besides oxidative modification, other post-translational modifications that regulate Nrf2 signaling include phosphorylation/dephosphorylation and acetylation/deacetylation [176, 177].

Besides its regulatory role in redox balance, Nrf2 has an overlapping but distinct role in ER stress responses. Nrf2 functions as a direct substrate of PERK that transduces UPR signal to the nucleus. PERK-Nrf2 pathway negatively regulates the CHOP-mediated apoptosis by inhibition of ATF4 binding to CHOP promoter [178]. PERK knockout was shown to interfere with protein synthesis that results in endogenous peroxide accumulation and increased level of pro-apoptotic ER stress marker CHOP [46, 179]. The resulting translational dysregulation could also be linked to impaired formation of stress granules (SG), which are non-membranous cytoplasmic foci that sequester nontranslating mRNAs with stalled translation initiation to promote cellular survival under stress conditions. The assembly of SG is mediated by redox balance between NAD(P)H and GSH, and has been implicated to suppress arsenite-induced ROS generation by controlling and coordinating the activities of two SG components, GTPase-activating protein SH3 domain binding protein 1 (G3BP1) and ubiquitin-specific protease 10 (USP10) [180, 181]. Under most circumstances, SG formation is dependent on the phosphorylation of eIF2α by PERK and other kinases [182]. Apart from direct phosphorylation of Nrf2 by PERK, Nrf2 can be activated through dimerization with ATF4, a canonical downstream factor of PERK [183]. Notably, Glover-Cutter et al. reported that SKN-1, the C. elegans functional ortholog of Nrf2, is regulated by ER sentinel proteins IRE1 and PERK, and controls transcriptional responses to ER stress [184]. In keeping with this finding, IRE1 was shown to trigger p38 MAPK-mediated antioxidant response driven by Nrf2 [9]. JNK is also capable of phosphorylating and activating Nrf2, indicating the potential involvement of IRE1-TRAF2-JNK pathway in the regulation of Nrf2. Moreover, Nrf2 is involved in the alleviation of proteotoxic stress by escalating pro-survival autophagy through phosphorylation of autophagy receptor SQSTM1/p62 when ubiquitin–proteasome pathway activity is undermined. SQSTM1/p62 is a target gene of Nrf2, which forms a positive feedback loop contributing to the acquisition of resistance to proteasome inhibitors in MM cells [44, 185]. Collectively, these findings imply that Nrf2 plays a pivotal role in orchestrating redox homeostasis under the control of UPR signaling.

A recent study found constitutive expression of Nrf2 in MM cell lines and about 50% of the primary MM cells tested [186]. Interestingly, Wruck et al. reported that Nrf2 is a potent transcriptional activator of IL-6 that binds to the antioxidant response element sequence in the promoter region of IL-6 gene [187]. IL-6 is a growth and survival factor in MM that promotes osteoclastogenesis and modifies bone marrow microenvironment favorable to disease progression. In addition, genetic silencing of Nrf2 in proteasome inhibitor-treated MM cells exacerbates ER stress and oxidative stress through dysregulation of GSH generation and CHOP expression, thus resulting in increased cell death. This is consistent with other reports showing that genetic and pharmacologic inhibition of Nrf2 re-sensitizes drug-resistant myeloma cells to carfilzomib and bortezomib, supporting the notion that malignant plasma cells may hijack the cytoprotective Nrf2-mediated pathways to fuel MM progression and therapeutic resistance [188, 189].

## UPR and HIF underlying hypoxic adaption in multiple myeloma

It is well established that hypoxia, or insufficient oxygen supply, is a characteristic feature of tumor microenvironment driving cancer advancement and therapeutic resistance, which also holds true for MM residing in a hypoxic bone marrow niche [190]. Bone marrow is a special type of tissue with an unusually low-oxygen tension (pO_2_) and a heterogeneous distribution of pO_2_ ranging from 1–6% (~ 7–43 mm Hg) which provides a conducive condition for hematopoiesis [191]. Emerging evidence suggests that hypoxia encourages MM cell dedifferentiation and quiescence by reducing plasma cell-specific transcription factors (BLIMP-1, IRF4, XBP1), increasing B-cell gene expression signatures (PAX5, CD19, CD20 and CD45) and stem-cell markers (OCT4, NANOG, SOX2), as well as inducing reversible cell cycle arrest [192, 193]. Given the frequent occurrence of anemia in MM, it is plausible that alterations in the bone marrow microenvironment in MM may redirect the process of hematopoiesis to stem-cell maintenance while limiting the commitment of hematopoietic progenitors to differentiation and maturation. Furthermore, hypoxia can lead to myeloma cell migration to secondary sites in the bone marrow via adoption of the properties of epithelial-mesenchymal transition (EMT) [194].

Tumor cells that survive under hypoxic conditions elicit adaptive responses that entail a decrease in oxygen consumption and energy expenditure, and transcriptional reprogramming that maintains hypoxic tolerance, which mostly involve UPR and HIF pathways [195]. Differential activation of these hypoxic responses depends primarily on the magnitude and duration of hypoxia. PERK-eIF2α pathway activation under moderate hypoxia (0.5–1%) requires longer duration of exposure than in more severe hypoxia (< 0.05% O_2_) [196], whereas HIF-1α protein has lower activation threshold as compared to UPR and can be activated under mild and moderate hypoxia (< 2%) [197]. The functional importance of PERK as a cytoprotective mediator against hypoxia-induced cell death has been supported by previous studies showing that abrogation of PERK-eIF2α-ATF4 signaling in tumor cells (e.g., PERK^−/−^ mice cells expressing dominant-negative PERK allele or inactive eIF2α mutant) results in a decrease in cell viability and clonogenic capability under hypoxia [198–200]. PERK-dependent eIF2α phosphorylation has been found to be indispensable for the survival of a subpopulation of hypoxic tumor cells highly resistant to chemotherapy attributable to GSH synthesis induction and ROS defense [201]. Whether this is a universal mechanism applicable to MM requires further investigation. It was also reported that HIF-1α confers survival advantage to tumor cells under hypoxic conditions, whereas compromised HIF-1 signaling neither significantly sensitizes tumor cells to cycling hypoxic stress nor affects the emergence of radiation-resistant cells. This indicates that PERK and HIF may support hypoxic cell survival through different but related mechanisms. The interaction between UPR and hypoxia response pathways enhances transcriptional activation of HIF-1 and its target gene expression [202]. However, proteasome inhibitor-induced ER stress can suppress HIF-1α activity under moderate hypoxia by inhibiting HIF-1α mRNA translation via PERK pathway, the activation of which leads to the redistribution of RNA-binding protein Y-box binding protein 1 (YB-1) to stress granules that prevents it from associating with HIF-1α mRNA [203, 204].

IRE1-mediated XBP1 mRNA splicing serves as another major determinant of cellular response to hypoxia. Hypoxia induces activation of the IRE1 arm of the UPR and upregulation of XBP spliced protein [205]. XBP1 in turn regulates transcriptional response to hypoxic stress via colocalization with the HIF-1α at the common regulatory element as confirmed by CHIP-seq and motif analysis [206]. XBP1 deficiency impedes tumor growth by sensitizing tumor cells to hypoxic cell death with negligible impact on the expression and secretion of angiogenic growth factors, suggesting that XBP1 may directly contribute to tumor growth under hypoxic conditions that circumvents the necessity for angiogenesis [207]. Mimura et al. reported that IRE1α inhibitor MKC-3946 prevents XBP1 splicing and inhibits the growth of MM cells under hypoxic condition in vitro and in vivo using a murine xenograft model SCID-hu with a transplanted human bone marrow [15]. Unlike the pro-survival effects of PERK and IRE1 in the adaptation to hypoxia, ATF6 and ATF4 have been mainly implicated in the regulation of ER stress-induced apoptosis during hypoxia by increasing CHOP expression under the control of HIF-1α [208, 209].

Hypoxia induces ROS production despite insufficient oxygen supply, while ROS stabilize HIF-α by inactivating its negative regulator prolyl hydroxylase domain protein. The accumulation of HIF-α and subsequent binding to the dimerization partner HIF-β is a crucial step toward activation of the hypoxic adaptive response, which controls the expression of a broad spectrum of genes implicated in multiple aspects of cancer progression, such as cell survival, proliferation, erythropoiesis, angiogenesis, glycolytic metabolism, cell cycle regulation and redox homeostasis [210, 211]. Other studies have led to a new hypothesis, wherein ROS stimulate ERK and PI3K-AKT pathways and subsequently increase HIF-1α at the levels of transcription and translation through intermediate regulators such as mTOR, Ras-related C3 botulinum toxin substrate 1 (Rac1) and histone deacetylase (HDAC) [212]. High constitutive expression of HIF-1α and HIF-2α, which are positively correlated with bone marrow angiogenesis by upregulation of VEGF and stromal cell-derived factor 1 (SDF1), were observed in several MM cell lines and biopsy specimens from MM patients [213–216]. Raninga et al. reported that hypoxia induces NF-κB signaling by elevating TrxR1 protein levels in bortezomib-resistant MM cells, whereas knockdown of TrxR1 and redox protein thioredoxin 1 (Trx1) reverses drug resistance [217, 218]. HIF-1α overexpression can lend support to drug resistance in MM cells mediated by activation of NF-κB, ERK and PI3K-AKT [219, 220]. These signaling pathways are activated during both hypoxic and ER stress response, which supports the notion of signaling crosstalk between hypoxia and UPR in fine-tuning tumor cell adaptation and apoptotic pathways in MM [221].

## Mitochondria-associated ER membrane facilitating crosstalk between ER and mitochondria

**Fig. 2:**
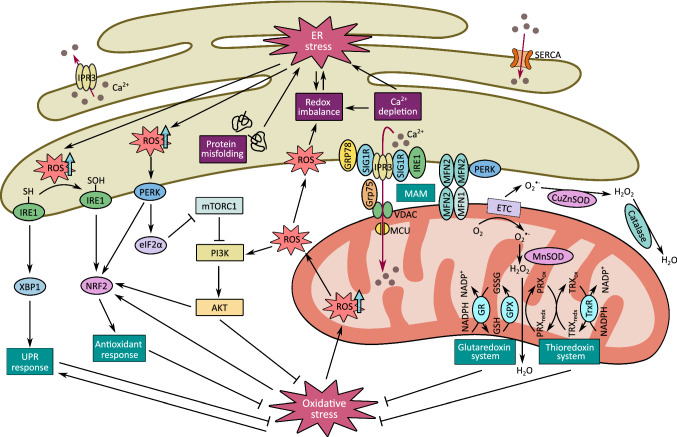
Crosstalk between UPR and redox signaling. ER stress occurs when ER Ca^2+^ is exhausted or ROS are overproduced due to cumulative load of protein misfolding and high energy demand. This in turn leads to redox regulation of UPR pathways. Cysteine oxidation of IRE1 drives a pathway switch to activation of Nrf2 and antioxidant responses. Redox-sensitive PERK also activates PI3K-AKT pathway to mitigate oxidative stress, while AKT may facilitate Nrf2 activation. IRE1 and PERK are key components of MAM for maintaining ER-mitochondria juxtaposition and ROS-dependent mitochondria apoptosis

Mitochondria-associated ER membrane (MAM) is a specialized subcellular entity formed by physical interaction between the ER and mitochondria that is closely linked to oxidative and ER stress responses (Fig. 2). MAMs are enriched with redox-sensitive calcium handling proteins, such as IP_3_R, sarco/ER Ca^2+^-ATPase (SERCA) pumps and modulators of mitochondrial Ca^2+^ uniporter (MCU), whose activities are controlled by regulatory proteins mediating Ca^2+^ flux dependent on local ROS concentrations [222–224]. A recent study revealed that one of the ER stress sensors, PERK, is an essential member of MAMs involved in Ca^2+^ signal transmission and ROS signal propagation from the ER to mitochondria during ER stress-induced apoptosis [225]. In addition, SERCA1 truncated variants (S1T) lacking Ca^2+^ pump activity are located at MAMs and these variants can be activated by PERK-eIF2α-ATF4-CHOP signaling pathway, which then induces apoptosis through mitochondria Ca^2+^ overload, increases MAM contact sites and promotes mitochondria immobilization [226]. IRE1 is also found to be associated with the MAMs-residing ER-chaperone sigma1 receptor (Sig-1R) that enhances IP_3_R-mediated mitochondrial Ca^2+^ uptake through dissociation from BiP upon depletion of ER Ca^2+^ and thereby counteracting ER stress [227]. Sig-1R facilitates activation of the IRE1-XBP1 pro-survival pathway by promoting IRE1 oligomerization during ER stress [228]. Aside from MAMs, peroxisomes have recently been found in close contact with the mitochondria and ER via inter-organelle tethering that enables coordinated regulation of fatty acid oxidation and lipid metabolism to maintain the balance between ROS and antioxidants [229, 230]. Interestingly, a genome-wide CRISPR/Cas9-based knockdown screening revealed that Ceapins, an inhibitor of ATF6α, induces inter-organelle communication between the peroxisome and ER through physical interaction between the peroxisomal transmembrane protein ATP-binding cassette sub-family D member 3 (ABCD3) and ER-resident ATF6α, thus inhibiting ATF6-mediated UPR signaling [231].

The functional roles of MAMs have been investigated in several types of solid cancer, such as breast, lung and prostate cancer, yet the activity of MAMs in hematologic malignancies remains to be elucidated [232]. Signaling crosstalk between the ER and mitochondria is suspected to play a role in cancer cell survival and stress tolerance in some hematologic disorders such as MM, AML and chronic lymphocytic leukemia, that display elevated levels of Bcl-2 family proteins involved in ER-mitochondrial Ca^2+^ dynamics [233]. Bcl-2 proteins have been observed at the MAMs. The BH3 domain of Bcl-2 protein interacts with pro-apoptotic proteins (e.g., Bim) and imposes inhibitory effect on apoptosis, whereas its BH4 domain interacts with IP_3_R isoforms, thus prevents Ca^2+^ flux into mitochondria and promotes cancer cell survival [234]. Recently, a decoy peptide Bcl-2-IP_3_R disrupter-2 (BIRD-2) targeted to the BH4 domain of Bcl-2 has been developed for cancer treatment. In vitro and in vivo studies have demonstrated that BIRD-2 propels IP_3_R-mediated Ca^2+^ signaling and causes pronounced apoptosis in Bcl-2 positive cancer models, including MM, follicular lymphoma and chronic lymphocytic leukemia [235]. Therefore, we may speculate that MAM and its associated membrane protein interaction may be a critical contributing factor in MM progression. Further investigations of this regulatory mechanism in the context of hematologic malignancies are warranted.

## Anti-myeloma therapeutic options targeting ER and oxidative stress

Over the past 15 years, substantial work has been dedicated to elucidating the biological and molecular mechanisms underlying MM pathogenesis, which have paved the way for the evolution of targeted therapeutic approaches. Several of these medications, including proteasome inhibitors, such as bortezomib, carfilzomib and ixazomib, HDAC inhibitor panobinostat and immunotherapies, such as immunomodulatory drugs (IMiDs) thalidomide and lenalidomide, monoclonal antibodies elotuzumab, daratumumab and the newly approved isatuximab, have been incorporated into treatment regimens as either mono- or combinatorial therapy [236]. The cytotoxicity of proteasome inhibitor, one of the first-line therapies for transplant-ineligible MM patients, is primarily attributable to amino acid deprivation, ERAD blockade and activation of PERK- and ATF6-mediated UPR pathways, which result in lethal ER stress and cytosolic oxidative damage due to mitochondrial dysregulation [51, 87, 237, 238]. It was considered that proteotoxic burden of undegraded proteasome substrates is underlying cause of proteasome inhibitor-induced cell death. However, there is increasing evidence that proteasome inhibition leads to lethal amino acid depletion, which can be rescued by amino acid supplementation while leaving the level of proteasome substrates unaffected [239]. Despite its demonstrated clinical success, many patients acquire tolerance to proteasome inhibitor and even develop cross-resistance to different proteasome inhibitors as the treatment proceeds, while some display intrinsic resistance due to relatively low proteasome activity [240]. The diverse sensitivity of MM cells to proteasome inhibitors can be explained by a prevailing model of proteasomal “load versus capacity”, which suggests that proteasome inhibitor-sensitive cells have a higher level of degradative workload and lower proteasome copy number and activity compared to their resistant counterparts [241]. In this regard, the short-lived rapidly degraded polypeptides (RDP), which are degraded within minutes after synthesis and may compete with endogenous ERAD substrates for proteasome-mediated degradation, accounts for a substantial fraction of newly synthesized proteins and imposes a significant workload on the ubiquitin–proteasome system [242, 243]. In MM cell lines and primary MM cells, a positive correlation has been observed between the responsiveness to proteasome inhibitor and the amount of polyubiquitinated proteins. The accumulation of polyubiquitinated proteins is accompanied by reduced availability of free ubiquitin under proteasomal stress in drug-sensitive MM cells. Taken together, the delicate balance between proteasomal capacity and functional workload could be exploited for therapeutic intervention against proteasome inhibitor resistance. This notion is supported by earlier findings indicating the synergistic cytotoxicity of ER stress-inducing drugs and proteasome inhibitors [244, 245].

**Fig. 3:**
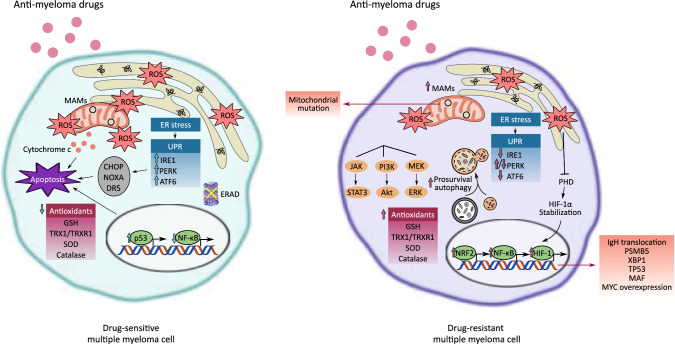
Differences between drug-sensitive and -resistant multiple myeloma cells. Anti-myeloma drug, such as proteasome inhibitor, induces apoptosis through multiple mechanisms, such as disruption of normal protein turnover by amino acid depletion, inhibition of ERAD, activation of UPR-mediated apoptosis signaling (e.g., CHOP, NOXA, DR5), induction of mitochondrial damage with increased Ca^2+^ transfer to the mitochondria at the MAM and reduction in antioxidant defense. Myeloma cells may acquire resistance through genetic mutations and/or transcriptional controls. This leads to downregulation of dependence on the UPR and upregulation of signaling pathways contributing to redox homeostasis, cellular survival and transition into quiescent state. *NOXA/PMAIP1* phorbol-12-Myristate-13-Acetate-Induced Protein 1, *DR5* death receptor 5

The adaptive resistance arises through various mechanisms, including mutations in the β5 subunit (PSMB5) of 20S proteasome [246], activation of NF-κB [247] and insulin-like growth factor-1 (IGF-1) signaling pathways [248], reduced dependence on UPR [27], upregulation of antioxidant response [249] and autophagic activity [250] (Fig. 3). The reader is referred to recent reviews on detailed resistance mechanisms of proteasome inhibitor [251, 252]. Hence, perturbation in redox homeostasis and mitochondrial energy metabolism may sensitize resistant cells to apoptosis and improve the efficacy of current treatment when used in combination with novel targeted agents. Therapeutic interventions targeting the UPR, particularly IRE1α/XBP1 and PERK, are in preclinical stages of development, as illustrated in Table 1. Notably, pharmacological inhibition of PERK may lead to pancreatic injury, a common side effect observed in animal models including mice, rats and dogs, and is ascribed to the other PERK function independent of ER stress response [253, 254]. Similarly, systematic disruption of IRE1α function may be accompanied by pancreatic exocrine dysfunction and/or hyperglycemia, which suggests that caution should be exercised when translating the experimental results of UPR targeting drugs into human clinical trials [255].

**Table 1:** List of promising anti-myeloma drugs targeting UPR & redox regulatory systems

Drug/treatment	Mechanism of action	Phase/status	References/identifier trial number (https://clinicaltrials.gov/)
ER-stress-inducing agents targeting UPR			
GSK2606414	Inhibits PERK, ↓ phospho-eIF2α	Preclinical	[263]
GSK2656157		Preclinical	[264]
MKC-3946	Inhibits IRE1α RNase activity, ↓ XBP1	Preclinical	[15]
STF-083010		Preclinical	[265]
Toyocamycin		Preclinical	[266]
4μ8C	Inhibits IRE1α RNase activity, ↓ XBP1 splicing, ↓ RIDD	Preclinical	[267]
CB-5083	Inhibits ERAD	Phase I/terminated	NCT02223598 [268, 269]
PAT-SM6	Inhibits GRP78	Phase I/completed	NCT01727778
ONC201	↑ phospho-eIF2α, ↑ ATF4, ↑ CHOP	Phase I/II/ongoing	NCT02863991
		Phase I/recruiting	NCT02609230
Sunitinib malate	↓ XBP1 splicing, ↓ VEGFR, ↓ PDGFR	Phase II/completed	NCT00514137
ROS-inducing agents targeting thioredoxin system			
Auranofin	Inhibits TrxR1/2, ↓ STAT3, ↓ NF-κB, ↑ ROS	Preclinical	[218, 270]
PX-12	Inhibits Trx1, ↓ HIF-1α, ↓ VEGF, ↑ ROS	Preclinical	[217]
ROS-inducing agents targeting glutaredoxin system			
Formononetin	↓ STAT3/5, ↓ GSH, ↑ GSSG, ↑ ROS	Preclinical	[271]
GO-203	↓ MUC1-C, ↓ TIGAR, ↓ NADPH, ↓ GSH, ↑ ROS	Preclinical	[109]
Imexon	↓ GSH, ↑ ROS, ↑ caspase-8/9	Phase II/completed	NCT00327249
As_2_O_3_ + AA	↓ GSH, ↑ ROS, ↓ NF-κB, ↑ JNK, ↑ p38 MAP kinase	Phase I/II/completed	[256]
As_2_O_3_ + AA + Bortezomib		Phase I/II/completed	[272]
As_2_O_3_ + AA + Bortezomib + Melphalan		Phase II/completed	NCT00469209
As_2_O_3_ + AA + Melphalan		Phase II/completed	NCT00661544
ROS-inducing agents targeting SOD and/or catalase			
Parthenolide	↓ Catalase, ↓ MnSOD, ↓ NF-κB, ↑ ROS	Preclinical	[273]
DSF	↓ CuZnSOD, ↓ ALDH1A1, ↓ Gli1/2, ↑ ROS	Preclinical	[259, 262]
Diethyldithiocarbamate (DDC) + Lanalidomide/Pomalidomide	↓ CuZnSOD, ↑ ROS	Phase NA/not yet recruiting	NCT04234022
Other ROS-inducing agents			
Sulindac	↑ p38 MAP kinase, ↑ ROS	Preclinical	[274]
Hypoxia-activated prodrug			
Evofosfamide (TH-302) + Dexamethasone ± Bortezomib	↓ cyclinD1/2/3, ↓ CDK4/6 ↓ p21^cip−1^, ↓ p27^kip−1^, ↓ pRb, ↑ caspase-3/8/9	Phase I/II/completed	NCT01522872

Selected preclinical and clinical studies

*VEGFR* Vascular Endothelial Growth Factor Receptors, *PDGFR* Platelet-derived growth factor receptor, *STAT3/5* Signal transducer and activator of transcription 3/5, *MUC1-C* Mucin 1 transmembrane C-terminal, *TIGAR* TP53 induced glycolysis regulatory phosphatase, *MnSOD* Manganese superoxide dismutase, *CuZnSOD* Copper-zinc-superoxide dismutase, *ALDH1A1* Aldehyde dehydrogenase 1 family member A1, *Gli1/2* Glioma-associated oncogene homologue ½, *CDK4/6* Cyclin-dependent kinase 4/6, *p21*^*cip−1*^ Cyclin-dependent kinase inhibitor 1, *p27*^*kip−1*^ Cyclin-dependent kinase inhibitor 1B, *pRb* Retinoblastoma protein

Encouraging preclinical results have been obtained when treating MM cells with ROS-inducing agents and other drugs that induce mitochondrial toxicity (Table 1). The cytotoxicity mechanisms of ROS-inducing agents involve dysregulation of different facets of antioxidant defense including the thioredoxin system, glutaredoxin system, SOD and catalase. Of these compounds, auranofin, arsenic trioxide (As2O_3_) and disulfiram (DSF), which are FDA-approved therapeutic options for other malignancies, are undergoing further research to be repurposed for treatment of MM. Auranofin and PX-12, a thioredoxin-1 inhibitor, have been shown to disrupt thioredoxin-mediated redox signaling and decrease the proliferation and clonogenicity of MM cells by inhibiting TrxR1/2 and Trx2, respectively.

In addition, depletion of intracellular GSH may account for increased cellular susceptibility to oxidative stress and reduced drug accumulation in target cells. GSH binds covalently to the trivalent form of arsenic, such as As_2_O_3_, and subsequently promotes drug extrusion through efflux pumps [256, 257]. In combination therapies containing GSH-depleting agents such as ascorbic acid, As_2_O_3_ has demonstrated beneficial clinical effects in inducing remission in MM patients, but As_2_O_3_ alone has limited efficacy. This implies that cellular GSH level plays a critical role in As_2_O_3_-induced apoptosis that impairs mitochondrial respiration and uncouples oxidative phosphorylation [258].

Inhibition of enzymatic antioxidant metalloprotein SOD, such as copper-zinc SOD (CuZnSOD/SOD1), using bivalent metal-ion chelator DSF and diethyldithiocarbamate (DDC) has been shown to enhance the cytotoxicity of proteasome inhibitors in drug-resistant cells [259]. Mice deficient in CuZnSOD were found to have heightened cellular oxidative damage and reduced GPX and mitochondria-resident manganese SOD (MnSOD) activities [260]. The formation of DSF/Cu complex suppresses MEK1 activation and induces apoptosis by restricting the availability of Cu to enhance MEK1 phosphorylation of ERK1/2 [261]. DSF and DDC are also known to inhibit aldehyde dehydrogenase (ALDH), a family of NADP-dependent enzymes associated with detoxification of reactive aldehydes, retinoic acid synthesis and drug tolerance, highly expressed in MM. DSF can eliminate a relapse-inducing subpopulation of ALDH^high^ stem-cell like MM cells by downregulating stemness transcription factors NANOG and OCT4 [262].

## Discussion and perspectives

Elevated ER stress response and aberrant redox status are classic hallmarks of MM cells. Emerging studies have provided promising insight into the intricate relationships between UPR, oxidative stress and survival pathways prominent in MM. Excessive production of immunoglobulin and several cytokines promotes ROS generation as a by-product of protein folding and mitochondrial energy metabolism, and activates numerous cytoprotective mechanisms that enable MM cells to evade from anti-myeloma therapies through elevation of ROS scavenging activities and further induction of UPR-related pathways. The discovery of regulatory nodes in the intracellular protein and redox homeostasis network and novel roles of ER stress sentinels may unveil potential anti-myeloma therapeutic targets to eliminate drug-resistant sub-clones while minimizing toxicity toward normal plasma cells.

Individualized therapeutic strategy focused on genetic and epigenetic basis of drug response variability among patients with MM may guide clinical decision making. The selective pressure imposed by treatments and the complex hypoxic tumor microenvironment may lead to the divergent evolution of quiescent tumor-initiating MM cells with drug resistance-conferring aberrations, necessitating an in-depth understanding of the evolution patterns of drug resistance for advanced development of therapeutic modalities against MM.

## Abbreviations

AKT: Activation of protein kinase B
AML: Acute myeloid leukemia
ASK1: Apoptosis signal-regulating kinase 1
ATF6: Activating transcription factor 6
BCR: B-cell antigen receptor
BiP: Binding immunoglobulin protein
CHOP: C/EBP-homologous protein
CHOP/GADD153: C/EBP-homologous protein
DUOX: Dual oxidase
eIF2α: Eukaryotic initiation factor-2α
ER: Endoplasmic reticulum
ERAD: ER-associated degradation
ERO1: Endoplasmic reticulum oxidoreductin-1
GSH: Glutathione
HIF: Hypoxia-inducible factor
IRE1α: Inositol requiring kinase 1α
JNK: C-Jun N-terminal kinase
MAM: Mitochondria-associated ER membrane
MGUS: Monoclonal gammopathy of undetermined significance
MM: Multiple myeloma
mTORC1: Rapamycin complex 1
NF-κB: Nuclear factor-κB
NOX: NADPH oxidase
Nrf2: BZIP Cap ‘n’ Collar transcription factor
PDI: Protein disulfide isomerase
PERK: Double-stranded RNA-activated protein kinase-like ER kinase
PI3K: Phosphoinositide 3-kinase
PRX: Peroxiredoxin
ROS: Reactive oxygen species
SMM: Smoldering myeloma
SOD: Superoxide dismutase
Trx: Thioredoxin
TrxR: Thioredoxin reductase
UPR: Unfolded protein response
XBP1: X‐box Binding Protein
